# COVID-19 in Pediatrics: Demographic, Clinical, Laboratory, and Radiological Characteristics of Infected Patients With SARS-CoV-2

**DOI:** 10.3389/fped.2021.808187

**Published:** 2022-01-13

**Authors:** Maryam Najafinejad, Fatemeh Cheraghali, Bahman Aghcheli, Abdolhalim Rajabi, Leila Barati, Hamed Naziri, Mohammad Hadi Gharib, Alijan Tabarraei, Britt Nakstad, Alireza Tahamtan

**Affiliations:** ^1^Department of Pediatrics, School of Medicine, Taleghani Children's Hospital, Golestan University of Medical Sciences, Gorgan, Iran; ^2^Department of Microbiology, School of Medicine, Golestan University of Medical Sciences, Gorgan, Iran; ^3^Department of Biostatistics and Epidemiology, Faculty of Health, Environmental Health Research Center, Golestan University of Medical Sciences, Gorgan, Iran; ^4^Department of Microbiology, School of Medicine, Guilan University of Medical Sciences, Rasht, Iran; ^5^Department of Radiology, School of Medicine, 5th Azar Hospital, Golestan University of Medical Sciences, Gorgan, Iran; ^6^Division of Paediatric and Adolescent Medicine, University of Oslo, Oslo, Norway; ^7^Department of Paediatrics and Adolescent Health, University of Botswana, Gaborone, Botswana; ^8^Infectious Diseases Research Centre, Golestan University of Medical Sciences, Gorgan, Iran

**Keywords:** children, COVID-19, SARS-CoV-2, Iran, Gorgan

## Abstract

The COVID-19 disease usually leads to mild infectious disease in children, but some develop serious complications. Here, we describe the characteristics of children with COVID-19 in northern Iran, the Golestan province. Ninety-one confirmed cases were enrolled in the study, aged 0–18 years. Demographic, clinical, comorbidity, laboratory, and radiological data were compared based on the disease severity (admitted to intensive care unit (ICU) or not) and disease outcome (recovered or deceased). Sixteen (17.5%) cases were hospitalized in ICU, and 8/91 (8.8%) deceased. Fever and cough were the most common clinical symptoms. Among all symptoms notified there were no significant differences between severe and milder cases, or between those who deceased and recovered. Failure to thrive (FTT), malignant disease and neurological disease were significantly more prevalent in severe cases as was frequently reported comorbidities. Laterality, ground-glass opacity, and lung consolidation were the most common findings in chest computed tomography. The data confirms that the COVID-19 disease has various presentations in children, and clinical, laboratory, and radiological findings may help predict the development of severe forms of COVID-19 among children.

## Introduction

A sudden outbreak of the novel severe acute respiratory syndrome coronavirus 2 (SARS-CoV-2) was identified in China at the end of 2019 and then spread worldwide (1). The World Health Organization (WHO) declared this a pandemic, the COVID-19 disease (2), a complicated respiratory disease that became a major global health threat (3). As of November 2th 2021, more than 247 million patients with COVID-19 have been confirmed globally, and more than 5.9 million confirmed cases have been reported in Iran.

Human beings are susceptible to the SARS-CoV-2 infection, though the clinical course and disease outcome vary from case to case (4). Patients with underlying diseases such as diabetes, hypertension, cardiovascular disease, and chronic bronchitis are the most affected and associated with much higher case-fatality rates (5). The severity of COVID-19 depends on a complicated interaction between the host, virus, and environment, leading to different clinical courses and disease outcomes (6, 7). Importantly, reported disease burden and case fatality rates differ considerably among different age groups (8, 9). In children, COVID-19 usually leads to a mild infectious disease, although some develop serious complications like the multisystem inflammatory syndrome (MIS-C) (10, 11).

The first reports on COVID disease indicated that children usually present with a mild or asymptomatic infection and are less prone to severe COVID-19 (12), maybe due to their staying at home during the pandemic with less contact with the source of infection, and a developing immune system with more pulmonary stem cells that can repair the injured cells (7). Some studies reported non-specific symptoms (13–15). Moreover, there is not enough data to determine the exact characteristics of SARS-CoV-2 infection in children. This paper reports demographic, clinical, laboratory, and radiological characteristics of pediatric COVID-19 cases admitted in Taleghani Children Hospital in Gorgan, north of Iran, and aims to define factors that may help predict the development of severe forms of COVID-19 among children, at least in northern Iran.

## Methods

This retrospective study was conducted on data collected from children hospitalized during March 2020 and February 2021 in a referral hospital for pediatric COVID-19, Taleghani Children Hospital, Gorgan, in the northern region of Iran. A total of 91 confirmed COVID-19 patients (aged 0–18 years) were enrolled in the study. All patients had been confirmed positive by reverse transcriptase Real-time PCR (rRT-PCR) targeting the SARS-CoV-2 nucleoprotein (N) and ORF1ab genes according to the Iranian national COVID-19 diagnostic protocol. Demographic, clinical, and laboratory data were collected from the patients' case reports. The available radiological findings [chest computed tomography (CT) images] were extracted from electronic medical records and were reviewed by a specialist. Patients were characterized and divided based on the following: disease severity [admitted to intensive care unit (ICU) or not] and disease outcome (recovered or deceased). The study was approved by the Ethics Committee of Golestan University of Medical Sciences (IR.GOUMS.REC.1400.142).

Statistical analyses were performed using SPSS version 25.0 (IBM Corp., Armonk, NY, USA). The continuous variables were presented as median (range), and the classification variables were presented in number (%). We performed the chi-square and Mann-Whitney tests to compare the continuous and classification variables between groups (ICU vs. non-ICU and recovered vs. deceased patients). *P* < 0.05 were considered statistically significant. As multiple hypotheses were tested, the likelihood of type I error increased. Data are available for Bonferroni-correction analyses for each individual hypothesis and presented in Tables.

## Results

Between March 14th 2020 and February 24th 2021, 91 children with confirmed COVID-19 (laboratory-confirmed cases) were admitted to the Taleghani Pediatrics Hospital in Gorgan city. Of 91 patients, 16 (17.5%) and 75 (82.5%) cases were hospitalized in ICU and non-ICU wards, respectively. Of these, 83 (91.2%) and 8 (8.8%) patients had recovered and deceased outcomes, respectively. Of all cases, 55 (60.4%) and 36 (39.6%) were males and females, respectively. The median age of all patients was 32 months (IQR 11–91). The patients were divided into different age groups, 33/91 were infants aged <1 year. No differences were observed in demographic data between groups. Details of demographic data are presented in Table 1.

**Table 1:** Demographic data of pediatric COVID-19 cases.

**Variables**	**Total**	**Outcome**		***P*-value**	**Severity**		***P*-value**
		**Deceased**	**Recovered**		**ICU**	**Non-ICU**	
Number (%)	91 (100)	8 (8.8)	83 (91.2)		16 (17.5)	75 (82.5)	-
Sex	
Male (%)	55 (60.4)	5 (62.5)	50 (60.2)	0.90	10 (62.5)	45 (60)	0.85
Female (%)	36 (39.6)	3 (37.5)	33 (39.8)		6 (37.5)	30 (40)	
Age (Months, Median, IQR)	32 (11–91)	27 (12–114)	32 (11–91)	0.59	21 (12–100.5)	34 (11–91)	0.85
Age groups (Months) (%)	
≤ 1	2 (2.20)	0 (0)	2 (2.41)	0.96	0 (0)	2 (2.7)	0.63
2–12	31 (34.06)	3 (37.5)	28 (33.73)		7 (43.75)	24 (32.43)	
13–36	17 (18.68)	2 (25)	15 (18.08)		4 (25)	12 (16.23)	
37–72	12 (13.19)	1 (12.5)	11 (13.25)		1 (6.25)	11 (14.86)	
>72	29 (31.87)	2 (25)	27 (32.53)		4 (25)	25 (33.78)	

*ICU, Intensive Care Unit; IQR, Interquartile Range; All deceased were admitted to ICU*.

Clinical symptoms apparent in the patient and signs that the physician perceived such as fever (68.13%) and cough (61.54%) were most frequently reported. There was a significant difference in dyspnea reported between recovered and deceased groups, as well as lethargy between the severe cases admitted to ICU vs. non-ICU cases of mild-to-moderate disease. Moreover, differences were found in immunodeficiency and neurological diseases between ICU and non-ICU cases. There were comorbidities such as Failure-to-Thrive (FTT), immunodeficiency, neurological diseases, and acute cardiac injury in both recovered and deceased groups. Totally, there were 8 deaths among our cases. The first who died of pediatric COVID-19 in our department was a 12 year old female with a history of dyspnea and aortic valve stenosis. Other expired female aged 5 months suffered severe failure-to-thrive (FTT). Two male fatal cases showed primary immunodeficiency disease. Another 2 male fatal cases with COVID-19 disease suffered from metastatic brain cancer and were immunosuppressed on chemotherapy treatment. One other male fatal case had gastrostomy feeding in cerebral palsy.

The underlying cause was unclear, anticipated as one several possible causes. Children aged <1 year were the prominent group of COVID-19 patients (33/91) of whom 3/33 deceased and 7/33 were severely ill and admitted to ICU. This infant group contributed to 3/8 of all who demised. The other major contributing group were children >6 years of whom 4/29 were admitted to ICU and 2 of these deceased. Four patients had co-infection, 2 with bacterial, Haemophilus influenza, and tuberculosis (TB), respectively. One was HIV positive and the fourth was seropositive for hydatid cyst. None of the co-infected patients died. Intubation was performed in about 10% of cases, all were ICU admitted. A significant number of deceased and ICU cases had been using corticosteroids. All acute respiratory distress syndrome (ARDS) cases were in deceased and ICU groups. Detailed information on age groups, deceased vs. recovered, ICU admitted with severe disease is given in Table 2.

**Table 2:** Clinical data of pediatric COVID-19 cases.

**Variables**	**Total**	**Outcome**		***P*-value**	**Severity**		***P*-value**
		**Deceased**	**Recovered**		**ICU**	**Non-ICU**	
Symptoms (%)^*^							
Fever	62 (68.13)	5 (62.5)	57 (68.67)	0.72	9 (56.25)	53 (70.67)	0.26
Cough	56 (61.54)	4 (5)	31 (37.35)	0.48	5 (31.25)	30 (40)	0.51
Dyspnea	27 (29.67)	5 (62.5)	22 (26.51)	0.03	8 (50)	19 (25.33)	0.05
Anorexia	27 (29.67)	3 (37.5)	24 (28.92)	0.61	3 (18.75)	24 (32)	0.29
Lethargy	24 (26.37)	1 (12.5)	23 (27.71)	0.35	1 (6.25)	23 (30.67)	0.04
Vomiting	21 (23.08)	3 (37.5)	18 (21.69)	0.31	4 (25)	17 (22.67)	0.84
Diarrhea	12 (13.19)	1 (12.5)	11 (13.25)	0.95	1 (6.25)	11 (14.67)	0.36
Distress	10 (10.99)	2 (25)	8 (9.64)	0.18	3 (18.75)	7 (9.33)	0.27
Myalgia	9 (9.89)	0 (0)	9 (10.84)	0.32	0 (0)	9 (12)	0.14
Abdominal pain	8 (8.79)	0 (0)	8 (9.64)	0.35	0 (0)	8 (10.67)	0.17
Skin rash	6 (6.59)	1 (12.50)	5 (6.02)	0.48	1 (6.25)	5 (6.67)	0.95
Comorbidities (%)^**^							
FTT^#^	30 (32.97)	6 (75)	24 (28.92)	0.008	7 (43.75)	23 (30.67)	0.31
Malignant disease	27 (29.67)	4 (50)	23 (27.71)	0.18	6 (37.5)	21 (28)	0.45
Immunodeficiency	17 (18.68)	5 (62.5)	12 (14.46)	0.001	7 (43.75)	10 (13.33)	0.005
Cardiac disease	3 (3.3)	1 (12.5)	2 (2.41)	0.12	1 (6.25)	2 (2.67)	0.46
Neurological disease	1 (1.1)	1 (12.5)	0 (0)	0.001	1 (6.25)	0 (0)	0.02
Acute cardiac injury	2 (2.2)	1 (12.5)	1 (1.20)	0.03	1 (6.25)	1 (1.33)	0.22
Medical Interventions (%)^***^							
Antibiotic	87 (95.6)	8 (100)	79 (95.18)	0.52	16 (100)	71 (94.67)	0.34
Oxygen with mask	19 (20.88)	3 (37.5)	16 (19.28)	0.22	5 (31.25)	14 (18.67)	0.26
Oxygen with hood	11 (12.09)	0 (0)	11 (13.25)	0.27	2 (12.50)	9 (12)	0.95
Intubation	9 (9.89)	6 (75)	3 (3.61)	<0.001	9 (43.75)	0 (0)	<0.001
Others (%)^****^							
Duration of hospitalization (days)	7	6.5	7	0.70	13	7	0.12
Contact with confirmed cases	10 (10.99)	0 (0)	10 (12.05)	0.29	1 (6.25)	9 (12)	0.50
Contact with suspicious cases	8 (8.79)	0 (0)	8 (9.64)	0.35	1 (6.25)	7 (9.33)	0.69
Hospitalization history	20 (21.98)	4 (50)	16 (19.28)	0.04	4 (25)	16 (21.33)	0.74
Corticosteroid drug user	6 (6.59)	3 (37.50)	3 (3.61)	<0.001	4 (25)	2 (2.67)	0.001
Co-infection	4 (4.4)	0 (0)	4 (4.82)	0.52	3 (18.75)	1 (1.33)	0.002
ARDS	6 (6.59)	6 (75)	0 (0)	<0.001	6 (37.50)	0 (0)	<0.001
Wheezing	30 (32.97)	6 (75)	24 (28.92)	0.008	10 (62.5)	20 (26.67)	0.006

*ICU, Intensive Care Unit; FTT, Failure To Thrive; ARDS, Acute Respiratory Distress Syndrome*.

*#Children are diagnosed with FTT when their weight or rate of weight gain is significantly below that of other children of similar age and sex. The pediatric patient may have more than one comorbidity*.

* *The threshold for significance is Bonferroni-corrected from P < 0.05 to P < 0.004*.

** *The threshold for significance is Bonferroni-corrected from P < 0.05 to P < 0.008*.

*** *The threshold for significance is Bonferroni-corrected from P < 0.05 to P < 0.01*.

**** *The threshold for significance is Bonferroni-corrected from P < 0.05 to P < 0.006*.

Laboratory data were statistically analyzed among all groups. The result revealed significant differences in parameters such as prothrombin time (PT), creatine phosphokinase (CPK), and lactate dehydrogenase (LDH) between recovered and deceased groups, and for white blood cells (WBC), PT, CPK, LDH, and aspartate aminotransferase (AST) between ICU and non-ICU groups. About 21% of all cases had lymphopenia, and 50% had elevated C-reactive protein (CRP) (≥ +1), though there was no significant difference between groups. Details of laboratory data are presented in Table 3.

**Table 3:** Laboratory data of pediatric COVID-19 cases.

**Variables**	**Total (Median, IQR)**	**Outcome**		***P*-value**	**Severity**		***P*-value**
		**Deceased (Median, IQR)**	**Recovered (Median, IQR)**		**ICU (Median, IQR)**	**Non-ICU (Median, IQR)**	
WBC (μ/l)	10,700 (6,500–16,000)	15,950 (7,150–23,700)	10,700 (6,300–14,800)	0.16	17,850 (9,600–23,000)	10,100 (6,100–13,600)	**0.004**
PMN (%)	60 (41.5–73.5)	62.5 (45–84)	60 (40–72)	0.47	66.5 (51–83.5)	59 (40–70)	0.09
Lymph (%)	39 (20.5–55)	35 (16–55)	39 (21.5–55)	0.59	25 (16.5–54)	39 (24.5–56)	0.16
HB (mg/dl)	10.85 (9.50–12.60)	10.40 (8.35–11.20)	11 (9.50–12.70)	0.15	11.2 (9.40–12.70)	10.85 (9.50–12.60)	0.94
PLT (mm^3^/μl)	185,000 (476–300,000)	107,000 (64,000–328,000)	191,500 (404–299,500)	0.70	107,000 (62,000–292,000)	191,500 (404–308,500)	0.87
ESR (mm/h)	20 (9–41)	15 (6–36)	20 (9–43.5)	0.50	25 (9–35)	19 (9–46)	0.71
BUN (mg/dl)	12 (9–14)	12 (10.5–16.5)	11 (9–14)	0.43	11 (9–13)	12 (9–14)	0.59
Creatinine (mg/dl)	0.6 (0.5–0.6)	0.6 (0.55–0.7)	0.6 (0.5–0.60)	0.28	0.6 (0.50–0.60)	0.59 (0.5–0.6)	0.61
K (mEq/L)	4.2 (4–4.8)	4.25 (3.65–4.7)	4.20 (4–4.80)	0.77	4.20 (4.10–4.70)	4.25 (4–4.80)	0.58
Na (mEq/L)	140 (137–143)	140 (135–143)	140 (137–143)	0.82	140.5 (138–143)	140 (137–142)	0.26
Ca (mg/dl)	8.7 (8.3–9.6)	8.3 (7.80–8.40)	9.05 (8.5–9.6)	0.20	8.40 (7.80–10)	9.05 (8.50–9.55)	0.42
INR (second)	1.2 (1–1.40)	1.70 (1.40–1.80)	1.10 (1–1.30)	0.04	1.40 (1.10–1.80)	1.15 (1–1.30)	0.26
PT (second)	14.10 (13.20–15.20)	17.35 (15.20–18.30)	13.80 (13–14.60)	0.006	15.85 (13.90–18.25)	13.80 (13–14.60)	0.02
PTT (second)	40 (38–46.5)	49.5 (37–70)	40 (39–40)	0.21	46.5 (39.5–60.50)	39.50 (38–40)	0.07
CPK (U/L)	144 (82–363)	938 (422–1,121)	125.5 (82–226.5)	0.01	570 (173–1,121)	106.5 (81–207.5)	0.004
LDH (U/L)	657 (559–883)	1366 (883–1,887)	650.5 (529.5–840.5)	0.006	1124.5 (731.5–1917.5)	651 (547–822)	0.01
ALT (IU/L)	36 (20–60)	56 (26–291)	35 (19–58)	0.12	52.5 (26–95)	35 (19–51)	0.13
AST (IU/L)	50 (37–71)	62 (49–333)	47 (35–71)	0.18	70 (43.5–166.5)	46 (35–60)	0.04
Abnormal urine analysis, N (%)	6 (6.59)	1 (12.50)	5 (6.02)	0.48	1 (6.25)	5 (6.67)	0.95
Lymphopenia, N (%)	19 (20.88)	3 (37.50)	16 (19.28)	0.22	5 (31.25)	14 (18.67)	0.26
^*^CRP positive, N (%)	45 (49.5)	4 (50)	41 (49.4)	0.97	9 (56.3)	36 (48)	0.54

*IQR, Interquartile Range; ICU, Intensive Care Unit; WBC, White Blood Cells; PMN, Polymorph nuclear leukocytes; HB, Hemoglobin; PLT, Platelet Cells; ESR, Erythrocyte Sedimentation Rate; BUN, Blood urea nitrogen; K, Potassium; Na, Sodium; Ca, Calcium; INR, International Normalized Ratio; PT, Prothrombin Time; PTT, Partial Thromboplastin Time; CPK, Creatine Phosphokinase; LDH, Lactate Dehydrogenase; ALT, Alanine Aminotransferase; AST, Aspartate Aminotransferase; CRP, C-reactive protein*.

* *One plus (+1) and higher are considered as CRP positive (a value of 8–10 mg/L or lower is normal)*.

*The threshold for significance is Bonferroni-corrected from P < 0.05 to P < 0.002*.

Of all patients, 22 cases had chest CT images available. The type and size of the pathological findings were used for classification. Laterality, ground-glass opacity, and lung consolidation were the most common findings in chest CT. However, significant differences were found in crazy paving pattern and pleural effusion between recovered and deceased groups. Details of radiological findings are presented in Table 4.

**Table 4:** Radiological findings of pediatric COVID-19 cases.

**Variables**	**Total (N:91)**	**Outcome**		***P*-value**	**Severity**		***P*-value**
		**Deceased (*n* = 8)**	**Recovered (*n* = 83)**		**ICU (*n* = 16)**	**Non-ICU (*n* = 75)**	
Chest CT (%)^*^	22	2	20	–	3	19	–
Laterality	22 (100)	2 (9.09)	20 (90.91)	0.995	3 (13.63)	19 (86.37)	0.577
Ground-glass opacity	22 (100)	2 (9.09)	20 (90.91)	0.955	3 (13.63)	19 (86.37)	0.577
Lung consolidation	14 (63.64)	2 (14.28)	12 (85.72)	0.430	3 (21.42)	11 (78.58)	0.681
Reticular nodular opacity	7 (31.81)	1 (14.2)	6 (85.8)	0.593	1 (14.2)	6 (85.8)	0.812
Crazy paving pattern	2 (9.09)	1 (50)	1 (50)	0.037	1 (50)	1 (50)	0.223
Pleural effusion	2 (9.09)	1 (50)	1 (50)	0.037	1 (50)	1 (50)	0.223
Cardiomegaly	1 (4.54)	0 (0)	1 (100)	0.755	0 (0)	1 (100)	0.642
Lung lobar (segmental) distribution^#^	21 (95.46)	2 (9.5)	19 (90.5)	0.738	3 (14.28)	18 (85.72)	0.371
1–2	4 (19.04)	0 (0)	4 (100)		0 (0)	4 (100)	
3–4	7 (33.35)	1(14.28)	6 (85.72)		2 (28.57)	5 (71.43)	
5	10 (47.61)	1 (10)	9 (90)		1 (10)	9 (90)	
Lung involvement	22 (100)	2 (9.09)	20 (90.91)	0.194	3 (13.63)	19 (86.37)	0.425
25%	6 (27.28)	0 (0)	6 (100)		1 (33.34)	5 (66.66)	
50%	8 (36.37)	0 (0)	8 (100)		0 (0)	8 (100)	
75%	7 (31.80)	2 (28.57)	5 (71.43)		2 (6.66)	5 (93.34)	
100%	1 (4.55)	0 (0)	1 (100)		0 (0)	1 (100)	

*ICU, Intensive Care Unit; CT, Computed Tomography*.

*Findings are characterized and reviewed by a specialist in radiology*.

*#Lung lobar (segmental) distribution means the number of involved lobes*.

* *The threshold for significance is Bonferroni-corrected from P < 0.05 to P < 0.005*.

## Discussion

This is a retrospective study on the clinical features of pediatric patients with COVID-19 from Iran. The WHO guidelines and the Iranian national recommendation for COVID-19 were considered for case identification (16). The percentage of deaths in COVID-19-associated pediatric patients in this study is dramatically higher than in other studies which reported no or rare fatality among children (17–19). The reasons for this may be the presence of children with comorbidities or a higher number of samples in our study compared with other studies. Consistent with previous studies in adults (7, 20), the findings showed that male children tended to be more susceptible to COVID-19. Although no statistical difference observed between gender in this study, a reduced vulnerability of females to the infection could possibly be genetic, hormonal, and immunological differences or higher expression levels of ACE2 in males (7). Most confirmed and severe cases of COVID-19 were observed in children aged <1 year which was similar to previous reports (21). This may be because they cannot wear masks and require specific protective measures.

Literature indicates that children may be asymptomatic or have mild or no clinical symptoms compared with adults (21, 22). We included hospitalized children and found similar to previous studies, that fever and cough were the predominant clinical characteristics of COVID-19 among children (17, 23), followed by dyspnea (29%), lethargy and weakness (26%), and vomiting (23%). Gastrointestinal manifestations included anorexia, nausea, vomiting, and diarrhea that could develop into more severe outcomes in pediatric patients (24). We found gastrointestinal symptoms to be incredibly higher than in adults in previous studies (25, 26). A correlation between COVID-19 disease and skin rashes has been described by Guan et al. (27) and 3 cases were previously reported by Duramaz et al. in pediatric patients with COVID-19 (28). In line with this we found cutaneous manifestations in six children. The role of COVID-19 in the appearance of skin rashes in pediatric patients is controversial and more investigations are needed to conclude if skin rash is a clinical sign of COVID-19 disease.

Comorbidities may increase the risk of developing severe and fatal pediatric COVID-19 disease (29, 30). In this study, as previously reported by Ogimi et al. (31), children younger than seven years with immunosuppressive diseases or underlying medical conditions were more vulnerable to COVID-19. In our study, comorbidities such as FTT and immunodeficiency were major risk factors for death. We found a significant difference in the outcome of COVID-19 disease among children with and without FTT. Also, similar to previous studies, there was a strong correlation between comorbidities and illness severity and mortality (32). Our findings showed that immunodeficiency, neurological disease, and acute cardiac injury were comorbidities associated with developing severe disease and a higher risk of fatal outcome. Importantly, neurological manifestations were noted as presenting symptom or complication in two our cases. One patient presented transverse myelitis and another was diagnosed with viral encephalitis with possible parenchymal hemorrhagic components.

It has been revealed that intubation does not affect infected patients' death rate or illness severity (33). In this study, 6 (75%) deceased and 9 (43.7%) ICU patients needed intubation and ventilatory support, consistent with results in adults (34). Similar to Soltani et al., a study from Iran, only 10 (10.9%) of patients had a history of contact with confirmed cases (17). However, these findings were extremely different from two other studies, which reported that 90% had close contacts with confirmed patients (12, 35). ARDS is a life threatening lung injury, breathing becomes difficult with poor oxygenation, and ARDS is associated with poor outcomes or an increased risk for fatality in children hospitalized with SARS-CoV-2 infection (36). In our pediatric cohort 6/91 (6.59%) presented with ARDS and needed ventilatory support, in contrast to Khoshnood et al. (37) where almost 23% of adults patients showed ARDS. The median number of hospitalized days was seven, less than previously reported in adults (38). Factors such as discharge and hospitalization criteria in different countries may justify this difference.

Despite the WHO recommendation (39), which states that antibiotic treatment is not necessary for mild/moderate COVID-19 disease, antibiotics were prescribed for most patients (95.6%) in this study, with no differences between groups. As shown in Table 2, only 2 patients proved to be co-infected with bacteria species, Haemophilus influenzae, and tuberculosis, respectively. One patient was HIV positive and the fourth was seropositive for hydatid cyst. A total of 8/91 COVID-19 infected children died, and all had co-morbidities, with immune deficiency (primary or treatment-induced) being the most important co-morbidity associated with mortality. In the beginning of the pandemic guidelines for treatment and healthcare of diseased children and surveillance systems were not in place in Iran leading to non-uniform treatment of the diseased children. Monitoring of trends of disease activity among children, and following up with important steps to mitigate disease such as closing schools and kindergartens came later in the pandemic.

Prolonged PT and elevated PT/INR (international normalized ratio) were detected significantly more often in deceased and ICU admitted children. In line with previous studies in adults and children (40, 41), the correlation between coagulation irregularities and the disease severity was confirmed, indicating that liver dysfunction can be used as a marker for predicting the severity of COVID-19 in children (42). In this study, a tendency for increased severity was seen among cases with elevated AST, similar to results from a meta-analysis (43). ICU admitted and deceased patients had a significantly higher level of CPK compared with non-ICU and recovered patients, respectively, in line with other and systematic literature reviews (44, 45). It has been well-documented that elevated CPK level is associated with the increased likelihood of a poor outcome (44). Adult patients with severe symptoms have higher LDH as an indicator for tissue damage (46). Similarly, we prove higher LDH levels in deceased vs. recovered children as well as ICU admitted vs. milder affected children.

CRP is used to assess inflammation in the body, and its role in combating infection has been determined (47). Our study demonstrated that almost half of patients had positive CRP, but inconsistent with reports in adult COVID disease (48), CRP elevation was not associated with disease severity or death. Various mechanisms have been proposed for lymphopenia in COVID-19 patients, including direct effects of the virus on lymphocyte recruitment, destruction of lymphatic organs, and the presence of inflammatory cytokine storm (49). The results presented here are similar to previous studies (21, 35, 50), and inconsistent with adults, as decreased lymphocytes cannot be considered a useful marker in pediatric COVID-19 patients. Common laboratory markers such as CRP and lymphopenia do not appear to be usable in the evaluation of severity of illness among children with COVID-19 disease; nevertheless, further investigations are needed to confirm the findings of this study.

Laterality, ground-glass opacity, and lung consolidation were the most common findings in chest CT. Serrano et al., reported that 90% of pediatric patients had ground-glass opacities (51). In contrast to a previous study from UK that reported no effusion (52), we found prevalence of pleural effusion among 2 patients. Due to the mild clinical manifestation in children during COVID-19, CT scan seems to be a more reliable method than chest X-ray. There is a concern about the increased risk of radiation exposure during CT scan that sometimes led to choosing other methods of imaging in children. Appropriate use of imaging and CT findings can improve early diagnosis and clinical management at different stages of the disease (53). The most common method for imaging to detect pneumonia in pediatrics with confirmed COVID-19 is the chest CT scan. Despite limitations due to the small number of CT findings, our results are in line with previous studies indicating that the chest-CT can be a powerful method for investigation of pneumonia in pediatric COVID-19, even before the appearance of clinical symptoms (54). Like published literature in both children and adults, laterality and ground-glass opacity were our main finding, although with less extensive clinical manifestations compared with adults (55, 56).

It is well-accepted that SARS-CoV-2 can infect all individuals of any age. According to previous studies, activation and intensity of inflammatory cytokine cascades are the leading cause of death in older people with COVID-19 (7). Early in the pandemic, fatality and severity of illness were likely underestimated in children compared with adults, due to the low prevalence of severe infection and asymptomatic cases, and/or school closure and lockdown of child-care centers (57). There might have been little virus load in the airways, and presence of anti-inflammatory hormones like melatonin, and decreased expression of ACE2 and TMPRSS2 receptors in child airways (22). Although most children with positive SARS-Co-2 tests are asymptomatic or have mild clinical presentations, special attention should be given to children with comorbidities who may develop severe disease and MIS-C (58).

All hospitalized, pediatric COVID patients in this region were admitted to our hospital because this is the only pediatric hospital in the Golestan province. However, the low sample size is a limitation of this study. The limited number of children made adequate comparisons between age and gender impossible, as well as comparisons between some other subgroups. Further, the mortality rate was not consistent with previous studies in children with COVID-19; therefore more studies are needed to explore these issues in pediatric COVID disease.

## Conclusion

A pandemic of COVID-19 is still ongoing worldwide, and contrary to previous perceptions, children have fallen victims in alarming numbers. Among children admitted to our hospital in northern Iran the main risk factor for death of COVID-19 disease was comorbidity. We may suggest radiologic proven laterality and ground-glass opacity and elevation of some biomarkers (CPK, LDH, and PT) as potential indicators for severe disease or fatal outcome. Although few children were admitted to the hospital, the high death rate is worrying and should lead to more comprehensive studies. Since we only included hospitalized cases and the number of patients was limited, this study may have overestimated patient mortality and morbidity rates. More studies on pediatric COVID disease are needed for an improved understanding of how and which children can develop a serious course. This may be preventive and predict which children may develop severe disease, and improve management and treatment of COVID-19 illness among individuals at greatest risk for severe disease and death.

## Data Availability Statement

The raw data supporting the conclusions of this article will be made available by the authors, without undue reservation.

## Ethics Statement

The studies involving human participants were reviewed and approved by Ethics Committee of Golestan University of Medical Sciences (IR.GOUMS.REC.1400.142). Written informed consent to participate in this study was provided by the participants' legal guardian/next of kin.

## Author Contributions

ATah, MN, and FC conceptualized and designed the study, drafted the initial manuscript, and reviewed and revised the manuscript. ATah and BN designed the data collection instruments, coordinated and supervised data collection, and critically reviewed the manuscript. BA, LB, HN, and ATab collected data and reviewed and revised the manuscript. MG collected and reviewed the radiological images. BA and AR carried out the initial analyses and reviewed and revised the manuscript. All authors approved the final manuscript as submitted and agreed to be accountable for all aspects of the work.

## Conflict of Interest

The authors declare that the research was conducted in the absence of any commercial or financial relationships that could be construed as a potential conflict of interest.

## Publisher's Note

All claims expressed in this article are solely those of the authors and do not necessarily represent those of their affiliated organizations, or those of the publisher, the editors and the reviewers. Any product that may be evaluated in this article, or claim that may be made by its manufacturer, is not guaranteed or endorsed by the publisher.

## Abbreviations

SARS-CoV-2: Severe acute respiratory syndrome coronavirus 2
WHO: World Health Organization
MIS-C: Multisystem inflammatory syndrome
rRT-PCR: Real-time PCR
CT: Computed tomography
ICU: Intensive care unit
FTT: Failure-to-Thrive
ARDS: Acute respiratory distress syndrome
PT: Prothrombin time
CPK: Creatine phosphokinase
LDH: Lactate dehydrogenase
WBC: White blood cells
AST: Aspartate aminotransferase
CRP: C-reactive protein.
